# Whole-cell fungal transformation of precursors into dyes

**DOI:** 10.1186/1475-2859-9-51

**Published:** 2010-07-05

**Authors:** Jolanta Polak, Anna Jarosz-Wilkołazka

**Affiliations:** 1 Department of Biochemistry, Maria Curie-Skłodowska University, Akademicka 19, Lublin, Poland

## Abstract

**Background:**

Chemical methods of producing dyes involve extreme temperatures and unsafe toxic compounds. Application of oxidizing enzymes obtained from fungal species, for example laccase, is an alternative to chemical synthesis of dyes. Laccase can be replaced by fungal biomass acting as a whole-cell biocatalyst with properties comparable to the isolated form of the enzyme. The application of the whole-cell system simplifies the transformation process and reduces the time required for its completion. In the present work, four fungal strains with a well-known ability to produce laccase were tested for oxidation of 17 phenolic and non-phenolic precursors into stable and non-toxic dyes.

**Results:**

An agar-plate screening test of the organic precursors was carried out using four fungal strains: *Trametes versicolor*, *Fomes fomentarius*, *Abortiporus biennis*, and *Cerrena unicolor*. Out of 17 precursors, nine were transformed into coloured substances in the presence of actively growing fungal mycelium. The immobilized fungal biomass catalyzed the transformation of 1 mM benzene and naphthalene derivatives in liquid cultures yielding stable and non-toxic products with good dyeing properties. The type of fungal strain had a large influence on the absorbance of the coloured products obtained after 48-hour transformation of the selected precursors, and the most effective was *Fomes fomentarius *(*FF25*). Whole-cell transformation of AHBS (3-amino-4-hydroxybenzenesulfonic acid) into a phenoxazinone dye was carried out in four different systems: in aqueous media comprising low amounts of carbon and nitrogen source, in buffer, and in distilled water.

**Conclusions:**

This study demonstrated the ability of four fungal strains belonging to the ecological type of white rot fungi to transform precursors into dyes. This paper highlights the potential of fungal biomass for replacing isolated enzymes as a cheaper industrial-grade biocatalyst for the synthesis of dyes and other commercially important products. The use of immobilized fungal biomass limits free migration of cells and facilitates their reuse in a continuous system for precursor transformation.

## Background

Investigations of environmentally friendly oxidations represent an important contribution to the development of sustainable processes. In this context, enzyme-catalysed oxidation reactions with air as co-substrate are of considerable current interest. These reactions are low-cost processes, which permit the use of non-toxic reagents in aqueous solvent systems. A majority of oxidative biotransformation processes involve metabolizing cells or isolated enzymes [1]. Fungal laccase, the main oxidase produced by many wood-rotting strains, catalyzes oxidation of a broad range of organic and non-organic substrates [2,3]. Laccase has the ability to mediate oxidative coupling reactions between aromatic compounds, producing new structures, including coloured products [4,5]. Therefore, the use of laccase as a biocatalyst may be an alternative to chemical synthesis of existing or new dyes. Laccase-mediated conversion of phenolic derivatives possessing amino substituents in the *ortho *position has been reported to yield dyes with the structure of phenoxazinone [6-9] and laccase-mediated conversion of Acid Blue 62 produced a novel azoanthraquinone dye [10].

The production and purification of laccase for industrial purposes is still too expensive to provide for a commercially interesting alternative to chemical synthesis. A cheaper way to utilize the enzymes for the synthesis of new compounds could be to use fungal cultures with a well-known ability to produce extracellular laccase. The use of whole cells offers the important advantage of simple, and hence low-cost, catalyst preparation. The use of biomass to synthesize dyes "in situ" could be an efficient way of producing colorants in mild conditions, especially, in terms of chemicals, pH, and temperature. The aim of this study was to examine the ability of four fungal strains to transform simple organic precursors into new colour compounds, which could be used as dyes. Transformation of 17 precursors, known as intermediates applied in the coloration of keratinous fibre [11,12] and cotton and wool knitted fabrics [13,14], was tested using fungal biomass immobilized on selected carriers. Transformation of one of them, 3-amino-4-hydroxybenzenesulfonic acid (AHBS), by a laccase purified from *Cerrena unicolor*, resulted in the biosynthesis of a novel phenoxazinone dye [7,9]. The use of fungal cultures to transform various chemical compounds has already been reported in several studies [15-17] and fungal biomass immobilized on Scotch-Brite™[18], alginate beads [19], and stainless steel sponge [20] was utilized for decolouration of textile dyes. This is the first paper on the use of immobilized white rot fungal strains for synthesis of dyes.

## Results and Discussion

To find a new catalyst for synthesis of dyes, the mycelium of white rot fungi with a well-known ability to secrete laccase was used for biotransformation of specific precursors to dyes. The precursors were selected from a list of commercially available chemicals, and the main criteria of selection were their chemical structure (benzene and naphthalene derivatives) and their price [21,22].

### Agar-plate screening test

Agar-plate screening of several different precursors, benzoic and naphthalene derivatives was performed using fungal biomass as a biocatalyst (Table 1). Among these precursors were chemicals consisting of amino-, hydroxy-, nitro-, sulfonic- and carboxy- substituents. The ability of the fungi to grow in the presence of the precursors and to transform the latter into coloured products was monitored during 14 days of the experiment. Colour products were formed during the first days of transformation in the case of the best precursors. In order to check the autoxidation ability of the precursors, the appearance of their coloured products without the presence of actively growing mycelium was monitored (control samples). In the case of six precursors, autoxidation processes of different intensity were observed (Table 2). Two of those precursors, AHNBS and 4AHNS, were rejected from further study because of a very rapid and very intensive autoxidation. In the case of the other four precursors (2,5DABS, DHN, 6AHNS, and 2A3HP), a very slight autoxidation was observed, and these precursors were further tested for their biotransformation by immobilized fungal mycelium.

**Table 1:** Characterization of precursors tested using the agar-plate screening test

Name of precursor	Acronym	Substituents	*Eo *(mV)	*Er *(mV)
***benzene derivatives***				
1,2-dihydroxybenzene	Cat	1-OH, 2-OH	548	285
aniline-2-sulfonic acid	A2SA	1-SO_3_H, 2-NH_2_	1013	-0.5
3-aminobenzenesulfonic acid	3ABS	1-SO_3_H, 3-NH_2_	968	5.4^a^
3-amino-4-hydroxybenzenesulfonic acid	AHBS	1-SO_3_H, 3-NH_2_, 4-OH	518	-103
2,4-diaminobenzenesulfonic acid	2,4DABS	1-SO_3_H, 2-NH_2_, 4-NH_2_	818	
2,5-diaminobenzenesulfonic acid	2,5DABS	1-SO_3_H, 2-NH_2_, 5-NH_2_	433	-356^a^
3-amino-4-hydroxy-5-nitrobenzenesulfonic acid	AHNBS	1-SO_3_H, 3-NH_2_, 4-OH, 5-NO_2_	339^a^, 579	-92
5-sulfosalicylic acid hydrate	5SSA	1-COOH, 2-OH, 5-SO_3_H	-	-
2-formylbenzenesulfonic acid sodium salt	2FBSA	1-SO_3_Na, 2-CHO	-	-
4,5-dihydroxy-1,3-benzene-disulfonic acid	dHBdSA	1-SO_3_Na, 3-SO_3_Na, 4-OH, 5-OH	914^b^	
2-amino-3-hydroxypyridine	2A3HP	2-NH_2_, 3-OH	647	144, 6.8
2-phenylphenol sodium salt tetrahydrate	2PP	2-ONa	790	
***naphtalene derivatives***				
2,7-dihydroxynaphtalene	DHN	2-OH, 7-OH	672	
6-hydroxy-2-naphthalenesulfonic acid sodium salt	HNSA	2-SO_3_Na, 6-OH	893	-144
4-amino-3-hydroxy-1-naphthalenesulfonic acid	4AHNS	1-SO_3_Na, 3-OH, 4-NH_2_	251	194,-1.4
6-amino-4-hydroxy-2-naphthalenesulfonic acid	6AHNS	2-SO_3_Na, 4-OH, 6-NH_2_	499	
5-amino-1-naphthalenesulfonic acid	ANS	1-SO_3_Na, 5-NH_2_	680	

Table summarizes the chemical structure of precursors in comparison with their oxidation (*Eo*) and reduction (*Er*) potentials. The potentials of the precursors were obtained at the scan rate 50 mV/s with the exception of dHBdSA, which was tested at the scan rate 25 mV/s. ^a ^very small peak, ^b ^scan rate - 25 mV/s.

**Table 2:** Colour and its intensity for products obtained after transformation of precursors during agar-plate screening tests

Precursor	Precursors autoxidation*	Colour of product	Intensity of products colour**			
			
			***TV7***	***FF25***	***AB123***	***CU139***
***precursors transformed by fungi***						
AHBS	**-**	orange	**4**	**4**	**4**	**4**
ANS	**-**	orange/red	3	2	**4**	2
2,4DABS	**-**	brown	1	2	**4**	3
dHBdSA	**-**	yellow/orange	1	3	**4**	2
Cat	**-**	brown	0	**4**	2	**3**
***precursors about low ability to autoxidation***						
2,5DABS	**-/+**	purple red	**4**	**4**	**4**	**4**
DHN	**-/+**	green/blue	**4**	**4**	**4**	**4**
6AHNS	**-/+**	purple/red	**4**	3	**4**	3
2A3HP	**-/+**	yellow/orange	1	**4**	3	**2**
***precursors about high ability to autoxidation***						
AHNBS	**+**	orange	**AO**	**AO**	**AO**	**AO**
4AHNS	**++**	red	**AO**	**AO**	**AO**	**AO**
***precursors non-transformed by fungi***						
A2SA	**-**	none	0	0	0	0
3ABS	**-**	none	0	0	0	0
5SSA	**-**	none	0	0	0	0
2FBSA	**-**	none	0	0	0	0
HNSA	**-**	none	0	0	0	0
2PP	**-**	none	0	0	0	0

The ability of four fungal strains, *Trametes versicolor *(*TV7*), *Fomes fomentarius *(*FF25*), *Abortiporus biennis *(*AB123*), and *Cerrena unicolor *(*CU139*), to transform seventeen precursors into coloured products was tested using agar-plate methods during 14 days of cultivation.

*Autoxidation: - no, -/+ - slightly, + high, ++ very high;

**Intensity of product colour: 0 lack of colour, 1 - 4 intensity of colour from light to strong; **AO **- coloured product of autoxidation

On the basis of the results obtained from the screening test it was observed that the tested fungal strains had the ability to grow in the presence of 1 mM precursors and to transform them into products of different colour, from yellow and red to green and blue (Table 2). Only in the presence of 2-phenylphenol sodium salt tetrahydrate (2PP), were the mycelia of four fungal strains unable to grow; however a new, red coloured product was formed on an old agar plug, which was used for plate inoculation, probably as the result of the presence of oxidizing enzymes in this fragment of the mycelium. 2PP was recognized as toxic for the tested fungal strains and as such was excluded from further experiments. Five precursors, A2SA, 3ABS, 5SSA, 2FBSA, and HNSA, were not transformed into coloured products by the actively growing mycelium of any of the tested strains and so were also eliminated from future tests (Table 2).

The colour intensity of the products obtained during the biotransformation of the precursors by fungi was analyzed after 7 days of cultivation. This intensity was expressed by different numbers; the value 0 expressed a lack of colour and the values in the range from 1 to 4 expressed a rising intensity of colour. Among the four tested strains, only *AB123 *and *FF25 *showed a very high potential for biotransformation of the different precursors (value 4). Three of the tested precursors, AHBS, 2,5DABS, and DHN were transformed by all the strains equally. Additionally in the case of ANS, 2,4DABS, dHBdSA and 6AHNS the most intensive colours of products were obtained after their biotransformation using strain *AB123*, whereas Cat and 2A3HP were transformed very efficiently by *FF25*. Strain *CU139 *showed a medium level of biotransformation efficiency (value 3) in the case of 2,4DABS, Cat and 6AHNS used as precursors. Strain *TV7 *showed a low level of biotransformation (values from 0 to 2) in the case of 2,4DABS, dHBdSA, Cat and 2A3HP tested as precursors for coloured product formation.

### Correlation between electrochemical behaviour of precursors and their transformation to coloured products

To find correlations between the degree of precursor transformation by fungi and its oxidation potential, cyclic voltammetry (CV) was done. All the tested compounds were characterized using CV at a graphite working electrode at pH 4.5. In most cases, they exhibited a well-defined oxidation peak (*Eo*) and also, for some compounds, a reduction peak (*Er*). Table 1 presents all obtained *Eo *and *Er *values. The precursors displayed oxidation peaks in the potential range from 251 mV (for 4AHNS) to 1013 mV (for A2SA) and reduction peaks in the range from -356 mV (for 2,5DABS) to 285 mV (for Cat) at a scan rate of 50 mV/s. Only precursor dHBdSA displayed an oxidation potential at a scan rate of 25 mV/s. Out of all the tested precursors, only two compounds (5SSA and 2FBSA) did not exhibit both the oxidation and the reduction peaks at scan rates of 25 to 200 mV/s. For the nine substances, the oxidation reaction was reversible, and during CV both the oxidation and the reduction peaks were measurable (Table 1).

**Figure 1:**
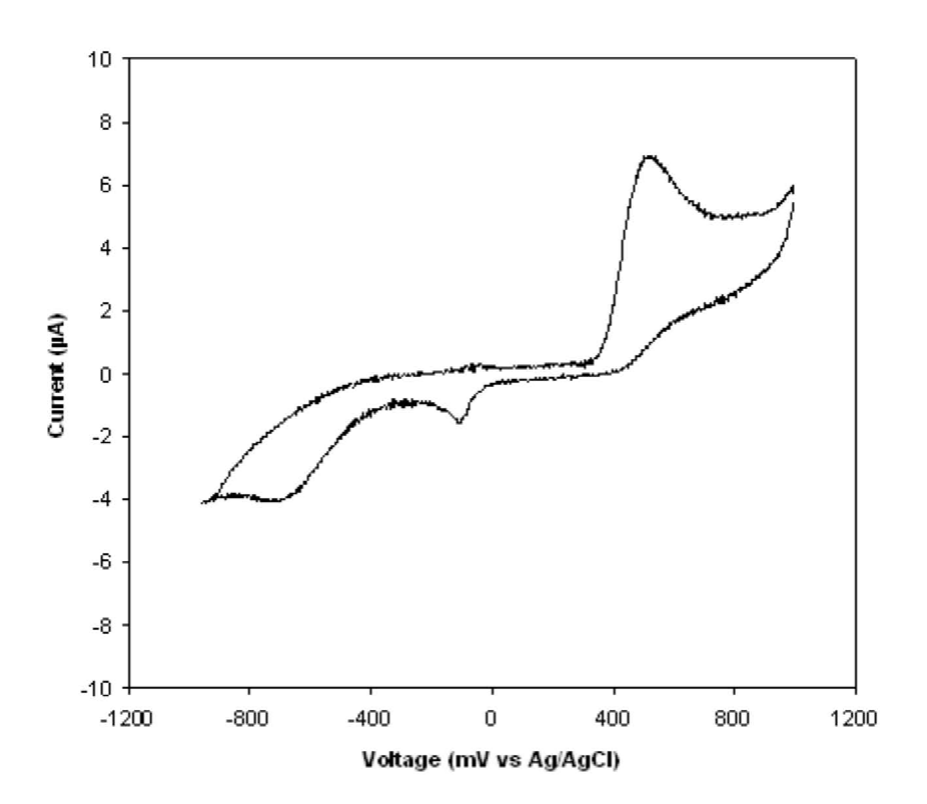
**Cyclic voltammograms of AHBS at graphite working electrode (Ag/AgCl reference)**. Solution containing 1 mM AHBS and 100 mM Na-tartrate buffer (pH 4.5) was scanned at a scan rate of 50 mV/s, first scan.

All the tested precursors (Table 1) possessed at least one of the following groups as substituents: -NH_2_,-COOH, -CHO and -OH, and there were just these groups that could be oxidized by laccase [23,24]. The precursors which were transformed by fungal laccase and simultaneously did not exhibit a high autoxidation level displayed the potentials which were comparable with the values of redox potentials of fungal LACs in the range from 500 to 800 mV [23]. Among these precursors were compounds belonging to dihydroxy- (Cat, DHN), amino- (ANS), or diamino- (2,4DABS, 2,5DABS) substituted compounds or precursors containing both amino and hydroxy groups (AHBS, 2A3HP, 6AHNS). All those precursors were transformed by the tested strains into coloured products during agar-plate screening tests. The value of *Eo *for the mentioned precursors ranged from 433 mV (2,5DABS) to 680 mV (ANS), and these precursors were transformed into intensively coloured products. The precursors with very high autoxidation ability (4AHNS and AHNBS) demonstrated oxidation peaks at 251 mV and 339 mV, respectively. Substrates with the value of *Eo *near 1000 mV (A2SA, 3ABS, and HNSA) and substrates which did not exhibit any value of redox potential (2FBSA and 5SSA) were not oxidized by the tested fungal strains. In the case of nine precursors, the presence of a reduction peak was noted (Table 1). Figure 1 presents a cyclic voltammogram of AHBS as an example of quasi-reversible behaviour with an oxidation potential of 518 mV and a small reduction potential of -103 mV.

### Transformation in liquid cultures

As a next step of this work, liquid cultures were tested to select (1) the best fungal strains for transformation, (2) the best carrier for fungal biomass immobilization, and (3) the best precursor for its possible transformation into coloured products. Only five precursors selected during agar-plate screening were tested using liquid cultures, and these included 2,5DABS, AHBS, 2A3HP, ANS, and DHN.

#### Immobilization of fungal biomass

**Figure 2:**
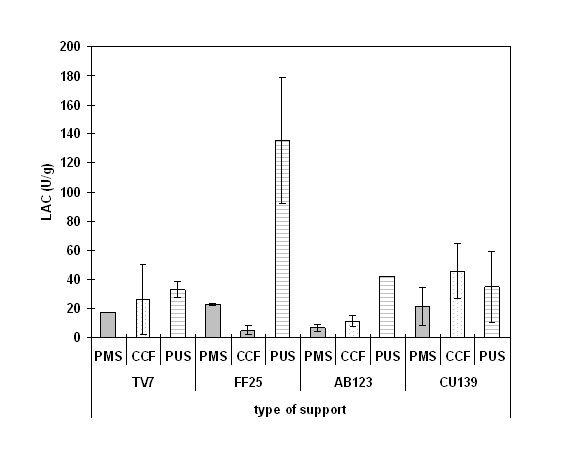
**Influence of the type of fungal biomass immobilization on laccase secretion by the tested strains**. Fungal biomass of four fungal strains, *Trametes versicolor (TV7)*, *Fomes fomentarius (FF25)*, *Abortiporus biennis (AB123)*, and *Cerrena unicolor (CU139)*, was immobilized on plastic mesh scourer (PMS), cellulose cook filter (CCF) and polyurethane sponge (PUS). The activity of extracellular laccase (LAC) was recorded on the 7^th ^day of cultivation of the four fungal strains.

To find the best support for immobilization of fungi, cheap and easy to handle supports, such as plastic mesh scourer (PMS), cellulose cook filter (CCF), and polyurethane sponge (PUS) were tested, to check the ability of white rot fungi to overgrow the support and to secrete LAC. Four fungal strains with a well-known ability to produce extracellular laccase [25-28] were used in the immobilization experiment. All the tested strains were able to adhere to the supports just after 1 day of cultivation and tightly overgrow their structure during the next 5-7 days. Only in the case of PUS, was the mycelium of all the tested strains unable to overgrow the support tightly and consequently many free pieces of mycelium were observed in the medium during the cultivation period. Figure 2 presents the activity of extracellular LAC (U/g) recorded on the 7^th ^day of cultivation for mycelium immobilized on PMS, CCF, or PUS as a support. It was observed that the activity of extracellular LAC secreted by mycelia of *TV7*, *FF25*, and *AB123 *immobilized on PUS was higher in comparison to the activity of LAC secreted by strains immobilized on PMS and CCF (Figure 2). The activity of LAC secreted by non-immobilized mycelia of the four tested strains was on average 50 times higher (data not shown) in comparison to the mycelia immobilized on the PMS and CCF supports. The obtained data confirmed the low ability of immobilized mycelium to secrete LAC [28]. However, the use of immobilized biomass limited free migration of cells and facilitated its reuse in a continuous system for precursor transformation. The level of laccase monitored in the immobilized fungal cultures was sufficient to apply this system for transformation of precursors into coloured products. Taking into account the results of this step of the experiment, the strains immobilized on plastic mesh scourer (PMS) were used for precursor biotransformation in liquid cultures.

#### Transformation of precursors and preliminary characterization of products

To find out which fungal strain was the best biocatalyst for dye synthesis, biotransformation of precursors 2,5DABS, AHBS, 2A3HP, ANS, and DHN was carried out using liquid cultures of immobilized biomass of the four fungal strains. These precursors were selected during the agar plate screening test. The transformation of the precursors was monitored using UV-vis absorption spectroscopy, and the maximum absorbances of the obtained products were determined based on a UV-vis spectrum in the range from 200 to 700 nm. The amount of product obtained during 48 hours of transformation was expressed as absorbance at a maximum monitored in UV-vis spectroscopy (Table 3).

**Table 3:** Maximum absorbance of products obtained during 48 hours of whole-cell biotransformation of selected precursors

Precursor	Wavelength of product λ_max _(nm)	Absorbance of product (Abs)			
		
		*TV7*	*FF25*	*AB123*	*CU139*
**2,5DABS**	**460, 544**	5.40 ± 0.05	**6.89 ± 0.03**	5.18 ± 0.02	3.24 ± 0.43
**AHBS**	**436**	5.94 ± 0.11	**8.62 ± 0.26**	5.84 ± 0.06	3.37 ± 3.49
**2A3HP**	**420**	4.79 ± 0.18	**7.85 ± 0.00**	6.39 ± 0.07	3.61 ± 1.69
**ANS**	**490**	**0.71 ± 0.02**	**0.70 ± 0.00**	**0.71 ± 0.04**	0.39 ± 0.14
**DHN**	**590**	0.18 ± 0.03	**1.00 ± 0.00**	0.87 ± 0.10	0.66 ± 0.39

Biotransformation of selected precursors was carried out in liquid cultures of four fungal strains (*TV7*, *FF25*, *AB123*, and *CU139*) immobilized on PMS. Absorbance of products was monitored spectrophotometrically after 48 hours.

**Figure 3:**
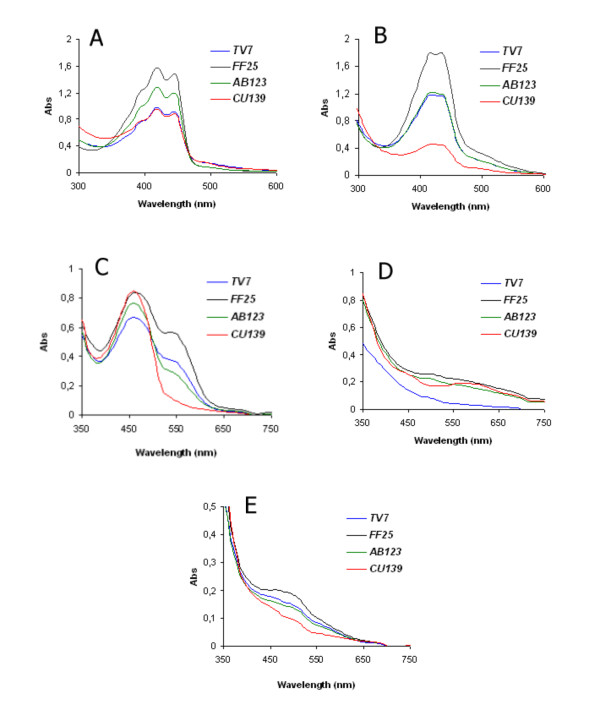
**UV-vis spectra of products obtained after transformation of selected precursors**. Whole-cell biotransformation of precursors 2A3HP (A), AHBS (B), 2,5DABS (C), DHN (D), and ANS (E) was performed in liquid cultures of four fungal strains (*TV7*, *FF25*, *AB123*, and *CU139*) immobilized on PMS.

After transformation of 2A3HP and AHBS by fungal biomass, yellow-orange coloured products were obtained. UV-vis spectra of those products showed a double peak at the wavelengths of 420 to 440 nm (Figure 3A, 3B). Fungal-mediated conversion of AHBS yielded a product with a typical UV-vis spectrum characteristic of phenoxazinone-type compounds, and laccase was shown to be responsible for this biotransformation [6-9,29-32].

In the case of 2,5DABS, it was observed that in the presence of fungal biomass, a slightly coloured medium containing the precursor turned into a red product after a very short time of cultivation. The UV-vis spectrum of the obtained product strongly depended on the pH, and the absorbance for this product was determined for two different wavelengths (460 nm and 544 nm), afterwards presented as the sum of absorbances at these two wavelengths (Figure 3C). A similar product, described as poly(2,5-diaminobenzenesulfonate), was obtained after transformation of 2,5DABS by horseradish peroxidase but with the necessity of adding hydrogen peroxide [33].

Three precursors, 2,5DABS, AHBS, and 2A3HP, were transformed into products with very intensive colours, whereas the intensity of the products obtained after DHN and ANS transformation was very low. In the case of the DHN precursor, the green-blue product of its transformation displayed a maximum at 590 nm (Figure 3D). The red product of ANS transformation had a maximum at 460 and 490 nm (Figure 3E), in contrast to a product obtained using chemical transformation of ANS described by Patel and Dasoni [34] with a maximum at 370 nm.

The maximum absorbance of products was determined during 48 hours of transformation (Table 3). Out of the five tested precursors, four yielded maximum amounts of products after transformation by *FF25*, with the exception of the precursor ANS, which was transformed at nearly the same ratio by three other strains (*TV7*, *FF25*, and *AB123*). In the liquid cultures, the lowest absorbances of products were obtained after transformation of all the tested precursors by strain *CU139*, contrary to the agar-plate screening test, in which *TV7 *demonstrated the lowest transformation level for most of the tested precursors.

#### Influence of pH

An extremely important factor determining the yield of enzymatic biotransformation of aromatic precursors is the value of the reaction mixture pH, which influences the catalytic activity of enzymes, precursor redox potential, and ionization of precursor substituents [35,36]. Usually the first step in enzyme-assisted catalysis is to optimize its parameters, among them the value of the pH. Generally, in enzyme biocatalysis, the pH is adjusted to a suitable value prior to the initiation of the reaction and in some cases it is controlled over time.

In this work, different reaction mixtures consisting precursors suspended in GPA medium with a specific pH were tested in liquid cultures. During the transformation of the selected precursors by fungal biomass it was observed that the tested strains adjusted the pH of the reaction mixture to the specific values characteristic of each strain (Table 4). Visible differences between the initial value of pH and the value of pH after 7 days of cultivation were observed for all the fungal strains. Only in *CU139 *cultures the pH values of the transformation mixture increased to basic (pH 8.3), whereas in *TV7 *cultures the pH values decreased to more acidic (pH 4.8) ones both in the solutions with the addition of precursors and in controls without their addition. The two remaining strains, *FF25 *and *AB123*, had the ability to adjust the pH values of the transformation mixtures to levels within a range from 5 to 6.

**Table 4:** The ability of the tested strains to modify the pH of the transformation medium during cultivation

Strain	2A3HP	2,5DABS	ANS	AHBS	DHN	Control I (without precursor)
	***initial pH***					
	**8.6**	**6.9**	**6.7**	**5.2**	**8.6**	**6.7**
	
	***after 24 hours***					
***TV7***	5.3	5.0	4.8	4.9	6	4.8
***FF25***	5.8	6.3	5.5	5.1	nd	5.0
***AB123***	5.0	5.2	5.2	4.9	5.6	5.0
***CU139***	6.5	6.7	6.2	8.0	7.0	6.7

**Control II** **(without strain)**	7.9	6.8	6.5	5.2	7.9	6.7

	***after 7 days***					
***TV7***	**6.5**	**4.6**	**4.6**	**4.8**	**6.0**	**4.8**
***FF25***	**6.0**	**6.4**	**6.2**	**5.4**	**nd**	**5.4**
***AB123***	**5.7**	**5.6**	**5.9**	**5.6**	**5.6**	**4.9**
***CU139***	**8.3**	**8.3**	**8.3**	**8.1**	**7.0**	**8.3**

**Control II** **(without strain)**	**7.5**	**7.0**	**6.8**	**5.2**	**7.9**	**6.7**

The pH values of transformation mixtures containing 1 mM precursors (2A3HP, 2,5DABS, ANS, AHBS, and DHN) were tested during 7 days of incubation with or without the presence of immobilized fungal biomass. Changes in the initial pH of the transformation mixtures (directly after precursor addition) were monitored after 24 hours and after 7 days of incubation in the presence of the four tested strains (*TV7*, *FF25*, *AB123*, and *CU139*). In addition the pH of culture liquids without a precursor (control I as biotic control) and without fungal biomass (control II as abiotic control) was monitored.

Fungi grow over a wide pH range, with the optimum pH in the weak acid range, which is connected with the natural role of oxidizing enzymes secreted by fungi involved in biodegradation of lignin [37]. Our study demonstrates the ability of fungi to modify the pH values of culture medium already after 24 hours of cultivation. Consistently with our results, Moreira Neto and co-workers described the ability of *Lentinus crinitus *and *Psilocybe castanella *to modify the pH value of the culture medium, which was found to be an important parameter for both the growth of the mentioned fungal strains and the enzymatic system involved in RBBR decolourization [38].

### Optimization of AHBS transformation by fungal biomass

Based on all the performed experiments and the obtained data, the precursor AHBS was selected for the next step of the study. AHBS was transformed by *FF25 *immobilized on plastic mesh scourer, which had been selected as the best biocatalyst and the best carrier, respectively.

**Figure 4:**
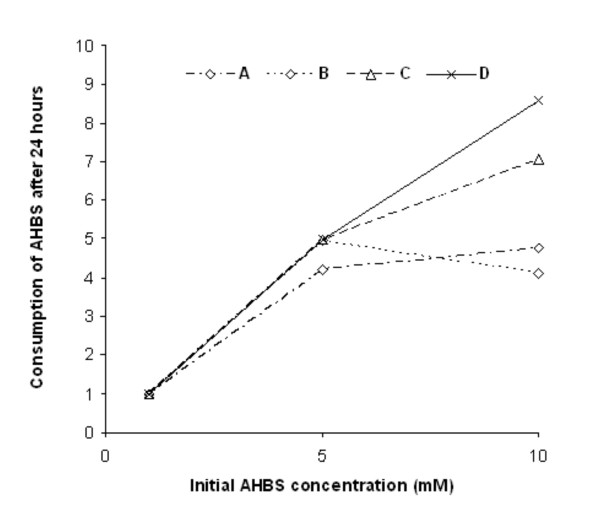
**Consumption of AHBS by strain *FF25***. Consumption of AHBS by *Fomes fomentarius *(*FF25*) was observed after 24 hours of incubation of three different AHBS concentrations (1, 5 and 10 mM) dissolved in four different transformation media: A - 1 mM Na-tartrate buffer, B - distilled water, C - 100 times diluted GPA medium, D - 10 times diluted GPA medium.

To find the yield of transformation of the precursor by *FF25*, three different concentrations of AHBS (1, 5, 10 mM) were incubated in four different transformation systems: system A - 1 mM Na-tartrate buffer, system B - distilled water, system C - 100 times diluted GPA medium, system D - 10 times diluted GPA medium. The consumption of the precursor over time was monitored using capillary electrophoresis. In the case of low precursor concentrations (1 mM and 5 mM), the type of system applied had no influence on the yield of AHBS transformation, and this precursor was transformed completely after 24 hours (Figure 4). The presence of 0.8 mM of AHBS in the reaction mixture after 24 hours of transformation was only observed for 5 mM AHBS incubated in Na-tartrate buffer.

**Figure 5:**
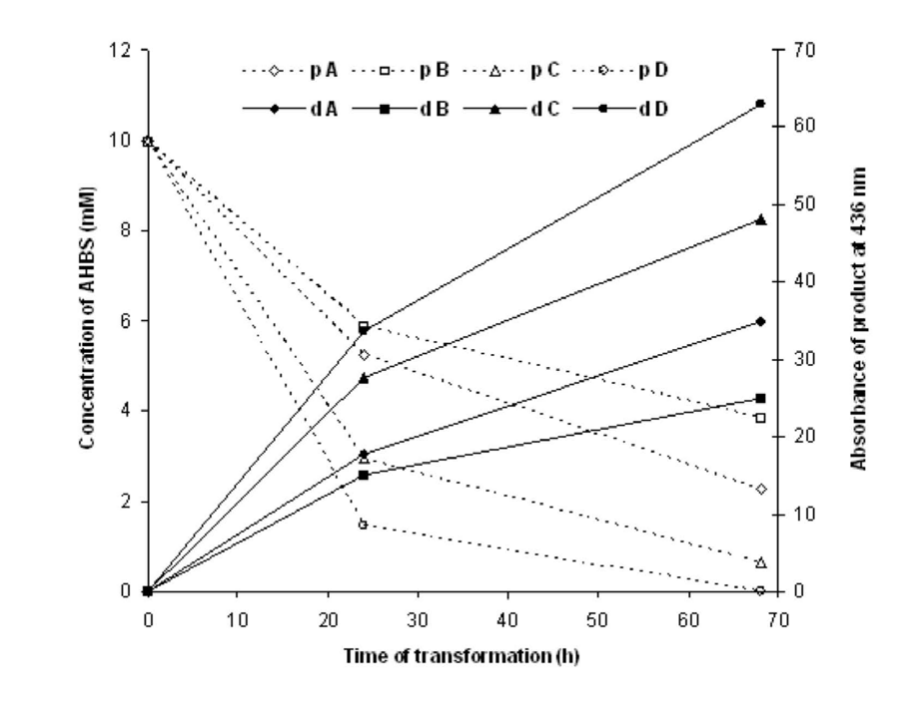
**Consumption of AHBS (p) and formation of the phenoxazinone dye (d) during 68 hours of transformation by strain *FF25***. Consumption of precursor was monitored using MEKC, whereas formation of product was monitored spectrophotometrically at 436 nm. Transformation of 10 mM AHBS was carried out in four different transformation media: A - 1 mM Na-tartrate buffer, B - distilled water, C - 100 times diluted GPA medium, D - 10 times diluted GPA medium. pA (pB, pC, pD) - concentration of precursor (AHBS) in system A (B, C, D). dA (dB, dC, dD) - absorbance of product (phenoxazinone dye) in system A (B, C, D)

The higher concentration of AHBS (10 mM) was not transformed as effectively as the lower one (5 mM) during 24 hours, regardless of the transformation system applied. More than 50% of AHBS concentration was transformed during 24 hours only in the case of both the 100 and 10 times diluted GPA medium (system C and D). These systems were the most effective for transformation of this concentration of precursor what was correlated with the highest absorbance of obtained product (Figure 5).

*FF25 *had the ability to transform 1 mM and 5 mM AHBS even when the strain was incubated in water or in buffer. However, the lack of nutrition over the long time of cultivation resulted in reduced biomass activity and transformation efficiency of the higher concentration of the precursor. The application of diluted GPA medium was preferable to water or to buffer. It was shown that the proposed system may be applied for the transformation of AHBS into a phenoxazinone dye, which had previously been described for the transformation of this precursor by a fungal laccase [7,9].

The use of fungal biomass for the transformation of precursors does not require adjustment of the pH by adding appropriate ions, thus offering several advantages. The application of laccase-secreting fungal biomass as a whole-cell biocatalyst can result, as is the case with natural degradation of lignin, in synchronized action of the intra- and extracellular oxidizing enzymes and low-molecular-weight fungal metabolites which could play a significant role as mediators in oxidation of a wide range of chemicals [39].

### Toxicity analysis of products

The cytotoxicity of precursors and products was tested using the Neutral Red uptake assay (NRU) with Caco-2 cells (Table 5). The EU classifies a chemical to be not environmentally toxic if the IC_50 _is above 0.1 mg/mL, which was the case for the selected products as measured with the NRU test. Among the five tested precursors, 2A3HP and DHN showed a significant toxicity, IC_50 _= 0.055 mg/mL and IC_50 _= 0.107 mg/mL, respectively, in contrast to dyes obtained from their transformation, which had IC_50_'s higher than 1 mg/mL, which is equivalent to very low toxicity. The dye obtained after transformation of 2,5DABS using *FF25 *had an IC_50 _of 0.57 mg/mL, whereas two other samples of this dye, obtained after 2,5DABS transformation by *AB123 *and *CU139*, were non toxic (IC_50 _> 1 mg/mL). In the case of the dyes obtained after transformation of AHBS and ANS by *FF2*5, *AB123*, or *TV7*, all the samples had IC_50_'s of more than 1 mg/mL and were essentially non-toxic. In the case of control cultures, growing without the addition of precursors, only the sample of *TV7 *was slightly toxic (IC_50 _= 0.85 mg/mL) while other control cultures (*AB123*, *CU139*, and *FF25*) had IC_50_'s higher than one (Table 5).

**Table 5:** Results of NRU assay of cytotoxicity tests done for precursors and products of transformation mediated by fungal strains (*TV7*, *FF25*, *AB123*, and *CU139*)

Precursor	IC_50 _(mg/mL)		Strain used for biotransformation
		
	Precursor	Product	
**2,5DABS**	>1	0.57 +/-0.11	*FF25*
		
		>1	*CU139, AB123*

**DHN**	0.107+/-0.0036	>1	*CU139*

**AHBS**	>1	>1	*FF25, AB123*

**ANS**	>1	>1	*TV7, AB123*

**2A3HP**	0.055 +/-0.028	>1	*AB123*

## Conclusions

**Figure 6:**
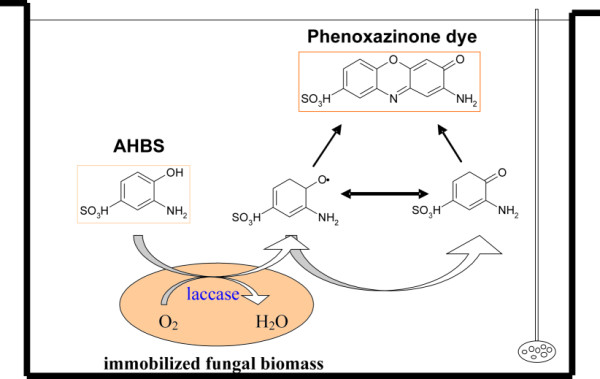
**Conversion of 3-amino-4-hydroxybenzenesulfonic acid (AHBS) by immobilized fungal biomass**. AHBS is oxidized to the phenoxy radicals and/or quinones, which can coupled non-enzymatically to coloured phenoxazinone compound [7].

This work is a preliminary attempt to estimate the capability of white rot fungi to be used in dye synthesis (Figure 6). Seventeen different precursors were tested as substrates for transformation by actively growing fungal biomass. Transformation of the selected precursors into intensive and non-toxic products was studied in liquid culture. The use of the fungi resulted in stable products lasting for a long time without specific stabilization. Further advantages included the simplicity of the process and the use of oxygen as a "clean oxidant". Further applicability of the fungi in the synthesis of new, coloured molecules is currently being investigated by our group. All the promising coloured products obtained in those investigations will be tested for their application as textile dyes as some preliminary results have already demonstrated that these products have valuable dyeing properties.

## Methods

### Chemicals

All the precursors were purchased from Sigma-Aldrich-Fluka Company and were used without further purification (Table 1). Stock solutions of the precursors were prepared in MiliQ water with or without the addition of sodium hydroxide for better solubilisation. The stock solutions of AHNBS, 5SSA, 2FBSA, and dHBdSA were prepared without the addition of NaOH (Millipore, USA), but in the case of A2SA, 3ABS, AHBS, 2,4DABS, 2,5DABS, 2A3HP, 2PP, HNSA, DHN, 4AHNS, 6AHNS, and ANS, the addition of NaOH was necessary for better solubilisation. The concentration of NaOH in precursor stock solutions did not exceed 1 mM. Catechol was the only precursor that required solubilisation by 50% ethanol, but the concentration of the alcohol in the biotransformation mixture did not exceed 1%. All the precursors were filter sterilized (Millipore, USA) and added to an appropriate sterilized medium to a final concentration of 1 mM. The structures of compounds with functional substituents are summarized in Table 1.

### Electrochemical potential of precursors

Cyclic voltammetry of the tested precursors was performed using an MTM-Anko apparatus type M161 (Poland) equipped with a graphite working electrode, a platinum counter electrode, and an Ag/AgCl reference electrode. The precursors were dissolved using 100 mM Na-tartrate buffer, pH 4.5, to 1 mM final concentration. All the precursors were tested at a scan rate of 50 mV/s; however, the precursors which did not display both the oxidation and the reduction potential at this scan rate were tested the scan rate varied from 25 to 200 mV/s (Table 1). All potentials in this work were referred to the Ag/AgCl reference electrode.

### Organisms and culture conditions

The fungal strains of *Trametes versicolor *(L. ex Fr.) Pil (*TV7*), *Fomes fomentarius *(L. ex Fr.) Kickx. (*FF25*), *Abortiporus biennis *(Bull. ex Fr.) Sing. (*AB123*), and *Cerrena unicolor *(Bull. ex Fr.) Murr. (*CU139*) were obtained from the Fungal Collection of the Biochemistry Department, Maria Curie-Sklodowska University (Lublin, Poland). The inoculation material was precultivated on 2% (w/v) malt extract agar (MEA) at 25°C. The biotransformation experiments, both in the micro-plate screening tests and in liquid cultures, were performed using modified potato medium (GPA). The composition of the GPA medium was as follows: 10 g/L glucose, 4 g/L potato extract, 1.5 g/L asparagine, and 1 g/L peptone; in the case of solid cultures, 20 g/L of agar was added to the medium.

### Immobilization of biomass

In order to select the best carrier for the immobilization of mycelium, three different inexpensive carriers were tested - plastic mesh scourer (PMS) (Stella, Poland), cellulose cook filter (CCF) (Vileda GMBH, Germany), and polyurethane sponge (PUS) (F.H. Lider, Poland), with an average weight of 1.15 g, 0.48 g, and 0.50 g per 100 mL of medium, respectively. The carriers were placed in 250-mL flasks containing 100 mL of the GPA medium. After autoclaving, the flasks were inoculated with 3 mL of 14-day-old mycelium homogenized using a laboratory disperser (IKA-Werke GmbH, Germany) and incubated at 28°C ± 2 on a rotary shaker at 140 rpm.

### Transformation of precursors

Biotransformation of the precursors by the fungal strains was performed using the solid cultures tested in the agar-plate screening test or using liquid cultures in Erlenmeyer flasks.

#### Solid cultures (agar-plate screening test)

The agar-plate screening test was performed using sterile 24-well tissue culture plates (16.2 mm in diameter). Each well contained 2.5 mL (± 0.2 mL) of sterile GPA medium with the addition of 2% agar and a precursor at a concentration of 1 mM. Each well was inoculated with one agar plug (3 mm in diameter) from the leading edge of colony, which had been maintained on MEA for at least 7 days at 25°C. The plates were incubated at 25°C, and the ability of the fungi to grow and transform the precursors into coloured products was monitored over the next 14 days.

#### Liquid cultures (Erlenmeyer flasks)

Liquid cultures were performed in 50-mL Erlenmeyer flasks containing fungal mycelium immobilized on plastic mesh scourer and 25 mL of GPA medium. After the carrier was overgrown by the mycelium, the cultivation fluid was decanted and the immobilized mycelium was poured over the biotransformation mixture containing 1 mM of precursor suspended in 10 times diluted GPA medium. Maximal absorbance of the products obtained during 48-hour transformation of the selected precursors was recorded at a specific wavelength characteristic of each product.

The pH of the biotransformation mixtures containing the tested precursors was not adjusted to a specific value in order to ensure sterility of the culture. The pH values of the transformation mixtures were tested directly after the addition of the precursors (initial pH, time 0), after 24 hours, and after 7 days of biotransformation.

### Optimisation of AHBS transformation by fungal biomass

Transformation of AHBS at concentrations of 1, 5, and 10 mM by strain *FF25 *immobilized on plastic mesh scourer was performed in 250-mL Erlenmeyer flasks. An appropriate amount of the precursor was dissolved in different transformation media: (A) 1 mM tartrate buffer, (B) distilled water, (C) 100 times diluted GPA medium, and (D) 10 times diluted GPA medium. The transformation was carried out for 48 hours in the case of 1 mM and 5 mM precursor concentrations and for 168 hours in the case of 10 mM AHBS in shaking cultures (140 rpm, 25°C). The consumption of AHBS during the transformation was determined using MEKC analysis at 24-hour intervals.

### Determination of laccase activity

The activity of extracellular laccase (LAC) was determined following oxidation of 2.5 mM 2,2'-azino-bis(3-ethylbenzthiazoline-6-sulphonic acid) (ABTS) in 100 mM Na-tartrate buffer at pH 3 [40]. The oxidation of ABTS was monitored spectrophotometrically at 414 nm (ε_414 _= 36 048 M^-1 ^cm^-1^). LAC activity was expressed in U per litre of culture fluid (U/L) or in U per 1 g of mycelium dry weight (U/g). One unit of LAC (U) oxidized 1 μmol of ABTS per 1 min at 25°C.

### Evaluation of precursor and product toxicity

The toxicity of precursors and products was evaluated using the Neutral Red uptake assay (NRU) with Caco-2 human intestinal cell-line as an *in vitro *test system [10]. Samples of products were lyophilized and then dissolved in dimethyl sulfoxide (DMSO) to a stock solution concentration of 100 mg/mL. The cytotoxicity of dyes was expressed as the half-maximal inhibitory concentration (IC_50 _- mg/mL), where values higher than 1 represented low cytotoxicity, values within the range from 0.1 to 1 represented medium cytotoxicity, and values below 0.1 represented high cytotoxicity of samples [10]. Additionally, the cytotoxicity of the fungal liquid culture without precursors and products was checked as a control.

### Micellar electrokinetic chromatography (MEKC)

MEKC analyses for optimisation of AHBS transformation by fungal biomass were performed on Thermo Capillary Electrophoresis, Crystal 100 (USA). The separations were carried out using fused silica capillary (80 cm, 50 cm to detection window, inner diameter of 50 Î¼m). The applied voltage was 29 kV, and the capillary temperature was maintained at 30°C. Samples were injected hydrodynamically for 1 s, and the detection was at 210 nm. The buffer solution used was 100 mM boric acid with 50 mM dodecyltrimethylammonium bromide (TTAB), and the pH was adjusted to 9.3 using NaOH. Liquid cultures after biotransformation by *FF25 *were prepared using MQ-water and filtered through 0.22 syringe filters before use.

## Competing interests

The authors declare that they have no competing interests.

## Authors' contributions

JP and AJW designed the research; JP performed the cultivation and transformation experiments, analyzed the data and drafted the manuscript. AJW conceived of the study, and participated in its design and coordination and helped to draft the manuscript. All authors have read and approved the final version of the manuscript.
